# Renal interferon-inducible protein 16 expression is associated with disease activity and prognosis in lupus nephritis

**DOI:** 10.1186/s13075-023-03094-8

**Published:** 2023-07-01

**Authors:** Xueyao Wang, Shaojie Fu, Jinyu Yu, Fuzhe Ma, Lihong Zhang, Jiahui Wang, Luyu Wang, Yue Tan, Huanfa Yi, Hao Wu, Zhonggao Xu

**Affiliations:** 1 Department of Nephrology, The First Hospital of Jilin University, Changchun, China; 2 Department of Renal Pathology, The First Hospital of Jilin University, Changchun, China; 3 Department of Pathology, Basic Medical College of Jilin University, Changchun, China; 4 Central Laboratory, The First Hospital of Jilin University, Changchun, China

**Keywords:** Lupus nephritis, Interferon-inducible protein 16, Biomarker, Clinicopathologic significance, Bioinformatics

## Abstract

**Background:**

Lupus nephritis (LN) is one of the most severe complications of systemic lupus erythematosus (SLE). However, the current management of LN remains unsatisfactory due to sneaky symptoms during early stages and lack of reliable predictors of disease progression.

**Methods:**

Bioinformatics and machine learning algorithms were initially used to explore the potential biomarkers for LN development. Identified biomarker expression was evaluated by immunohistochemistry (IHC) and multiplex immunofluorescence (IF) in 104 LN patients, 12 diabetic kidney disease (DKD) patients, 12 minimal change disease (MCD) patients, 12 IgA nephropathy (IgAN) patients and 14 normal controls (NC). The association of biomarker expression with clinicopathologic indices and prognosis was analyzed. Gene Set Enrichment Analysis (GSEA) and Gene Set Variation Analysis (GSVA) were utilized to explore potential mechanisms.

**Results:**

Interferon-inducible protein 16 (IFI16) was identified as a potential biomarker for LN. IFI16 was highly expressed in the kidneys of LN patients compared to those with MCD, DKD, IgAN or NC. IFI16 co-localized with certain renal and inflammatory cells. Glomerular IFI16 expression was correlated with pathological activity indices of LN, while tubulointerstitial IFI16 expression was correlated with pathological chronicity indices. Renal IFI16 expression was positively associated with systemic lupus erythematosus disease activity index (SLEDAI) and serum creatinine while negatively related to baseline eGFR and serum complement C3. Additionally, higher IFI16 expression was closely related to poorer prognosis of LN patients. GSEA and GSVA suggested that IFI16 expression was involved in adaptive immune-related processes of LN.

**Conclusion:**

Renal IFI16 expression is a potential biomarker for disease activity and clinical prognosis in LN patients. Renal IFI16 levels may be used to shed light on predicting the renal response and develop precise therapy for LN.

**Supplementary Information:**

The online version contains supplementary material available at 10.1186/s13075-023-03094-8.

## Background

Lupus nephritis (LN) is one of the most severe complications of systemic lupus erythematosus (SLE), an autoimmune disease that causes chronic, multiple-organ damage [1, 2]. Though the frequency of LN has decreased over the past several decades, up to 20% of LN patients progress to end-stage renal disease during the first decade of their illness [3]. Early diagnosis of LN and effective monitoring of disease activity are therefore crucial. However, the sensitivity and specificity of current conventional biomarkers for LN disease activity and chronicity, such as proteinuria amount, urine sediments, serum creatinine value, titers of anti-double-stranded DNA (anti-dsDNA) antibodies, and serum C3 levels, are restricted [4] The most accurate method for diagnosing LN and assessing the severity of kidney injury is still invasive renal biopsy [1, 5]. Histological indexes of LN, activity index (AI), and chronicity index (CI) defined by the National Institutes of Health (NIH) system have all made substantial contributions to the prediction of renal prognosis [6, 7]. However, the importance of the NIH indexes in portending long-term prognosis during initial kidney biopsy was not confirmed by the Lupus Nephritis Collaborative Study Group [8]. Therefore, it is necessary to explore more precise biomarkers that might help with LN early diagnosis and patient monitoring.

Recently, machine learning was developed to discover novel, potentially diagnostic biomarkers via differential gene expression. The accuracy of microarray for identifying differentially expressed genes (DEGs) has increased notably over past years [9, 10]. In the present study, we carried out bioinformatics analysis to identify potential diagnostic markers for LN. Using three separate machine learning algorithms, we identified interferon-inducible protein 16 (IFI16) as a biomarker for LN. Renal IFI16 expression was verified with immunohistochemical (IHC) staining. IFI16, a member of the interferon-inducible p200-protein family (PYHIN200 or HIN-200 protein family), is characterized by a 200-amino-acid motif containing a DNA binding domain at the C-terminus and a PYRIN domain at the N-terminus [11]. IFI16 may regulate a variety of biological processes, including cell differentiation, proliferation, senescence, apoptosis, and inflammasome assembly, according to researchers in the field of cellular biology [12–15]. It was reported that IFI16 could be regarded as a DNA sensor that is implicated in the innate immune response, particularly in defense against viral infections and activation of the interferon gene pathway [15–17]. IFI16 and systemic autoimmune disease have recently been connected by several lines of evidence. SLE [18], Sjӧgren’s syndrome [19, 20], systemic sclerosis [21] and rheumatoid arthritis [22] have all been linked to anti-IFI16 antibodies. IFI16 was detected in serum from patients with SLE, Sjӧgren’s syndrome, systemic sclerosis, rheumatoid arthritis and psoriasis [22–24]. Although aberrant IFI16 expression was closely associated with SLE, its potential as a disease biomarker for LN remains unclear. Moreover, there is no information on IFI16 expression in renal biopsies from LN patients.

To determine the significance of renal IFI16 expression in disease progression, we collected data from LN patients and analyzed the renal IFI16 expression in relation to the disease severity and the prognosis. To explore potential mechanisms of renal IFI16 in LN, we performed Gene Set Enrichment Analysis (GSEA) and Gene Set Variation Analysis (GSVA) as well as CIBERSORT to assess the relationships of IFI16 expression with immune infiltrating cells.

## Methods

### Identification of key biomarkers for LN using bioinformatics

Renal glomeruli microarray datasets for patients with LN and controls (GSE32591 and GSE99339) were obtained from the GEO database (http://www.ncbi.nlm.nih.gov/geo/) [25]. The 2 data sets were merged and used as training sets. The Combat function in the R sva package was utilized to correct inter-batch differences [26]. The R limma package was used to identify differentially expressed genes (DEGs) between patients with LN and controls [27]. Criteria for defining DEGs were adjusted *P*-value < 0.05 and |log fold change (FC)|> 1.5. Biological processes and pathways associated with DEGs were obtained using Gene Ontology (GO) and Kyoto Encyclopedia of Genes and Genomes (KEGG) enrichment analysis, respectively [28]. The analyses were performed with the clusterProfiler package in R. An adjusted *P*-value < 0.05 was regarded as statistically significant [29]. R packages RandomForest [30], e1071 with fivefold cross-validation [31], and glmnet [32] were used to conduct random forest (RF), support vector machine-recursive feature elimination(SVM-RFE) and least absolute shrinkage and selection operator(LASSO) logistic regression modeling, respectively, of disease status. Genes detected by all 3 machine learning algorithms were chosen as possible biomarkers. The GSE104948 data set was used to validate the prediction efficiency of identified gene biomarkers. Using mRNA expression data from the training and validation sets separately, a receiver operating characteristic (ROC) curve was constructed for the detected biomarkers. To evaluate diagnostic efficiency, the area under the ROC curve (AUC) was calculated. ROC curves were plotted with the R pROC package. A two-sided *P*-value < 0.05 was statistically significant [10].

### Patients and sample collection

One hundred four patients from the First Hospital of Jilin University were selected for IHC analysis between February 2012 to December 2020. Patients were diagnosed with SLE according to the 1997 American College of Rheumatology revised criteria and had biopsy-proven LN [33]. Renal disease control samples were obtained from 12 individuals with diabetic kidney disease (DKD), 12 with minimal change disease (MCD) and 12 IgA nephropathy (IgAN). Normal control (NC) tissue was collected from the renal tissues next to renal cancerous tissue from 14 patients with solitary renal cell carcinoma. These renal samples were identified as normal via regular light microscopy, immunofluorescence, and electron microscopy. Written informed consent was obtained from each patient. The research followed the Declaration of Helsinki and the design of this work was approved by the ethical committees of the First Hospital of Jilin University (approval number: 2022–552).

### Renal histopathology

Two independent pathologists assessed the renal histopathology of LN patients according to the International Society of Nephrology/Renal Pathology Society (ISN/RPS) classification system [5]. All patients enrolled in our study fulfilled the inclusion criteria of having at least 10 glomeruli in the renal biopsy, which is recommended as the appropriate number of glomeruli for evaluation [34]. Renal pathologists calculated pathologic parameters, including activity indices and chronicity indices, with a system that involved semiquantitative scoring of specific biopsy features as previously reported [5]. Endocapillary hypercellularity, neutrophils/karyorrhexis, fibrinoid necrosis, hyaline deposits, cellular-fibrocellular crescents, and interstitial inflammation were all activity indices. Total glomerulosclerosis score, fibrous crescents, tubular atrophy, and interstitial fibrosis were all considered chronicity indices.

### Clinical parameters

The clinical and laboratory data of LN patients, including age, sex, hypertension, fever, malar rash, oral ulcer, alopecia, arthritis, serositis, neurological disorder, nephrotic syndrome, SLE disease activity index (SLEDAI), leukocytopenia, thrombocytopenia, hematuria, leukocyturia, serum creatinine, serum C3, serum C4, albumin, 24-h proteinuria, and anti-dsDNA antibody, were extracted from the electronic medical records of the First Hospital of Jilin University.

For of renal outcome endpoints, conversion of anti-dsDNA antibodies from positive to negative and a ≥ 30% reduction from baseline estimated glomerular filtration rate (eGFR) were included. Serum creatinine level was used to calculate GFR according to the Chronic Kidney Disease Epidemiology Collaboration eGFR equation [35].

### Renal immunohistochemical (IHC) staining

Formalin-fixed paraffin-embedded renal sections from LN patients, disease controls and normal controls were deparaffinized in xylene and ethanol then rehydrated in graded ethanol. Slides were heated in 0.01 M citrate buffer pH 6.0 for 5 min to perform antigen retrieval. Freshly produced 3% H_2_O_2_ was utilized to quench the endogenous peroxidase activity for 10 min. Following an overnight incubation with the primary antibody (anti-IFI16 antibody, rabbit monoclonal, ab169788, Abcam, Cambridge, UK) at 4 °C, sections were treated for an hour with secondary antibodies (goat anti-mouse/rabbit, Maixin Company, Fuzhou, China) at room temperature. The 3, 3-diaminobenzidine (DAB) chromogenic substrate was used with the Ultra-Sensitive TM S-P detection system Kit (Maixin Company, Fuzhou, China) for visualization. Hematoxylin was used to subtly stain the slides and OLYMPUS cellSens Entry microscopy system was utilized for examination. The IHC staining results were evaluated by Image-Pro Plus (version 6.0; Media Cybernetics, Dallas, TX, USA) as the mean optical density (integrated option density/area). For quantitative assessments, all glomeruli in a section and 10, 40 × high power fields of tubulointerstitium per renal section were counted blindly.

### Multiplex immunofluorescence (IF) staining

Renal sections were heated for 5 min in either citrate buffer (0.01 M, pH 6.0) or in Tris/EDTA buffer (1x, pH 8.5) to retrieve antigens. Slides were then incubated with anti-IFI16 antibody (Abcam, Cambridge, UK), anti-Wilms Tumor protein antibody (anti-WT1, Abcam, Cambridge, UK), anti-CD31 (Maixin, Fuzhou, China), anti-integrin-ɑ8 (Anti-ITGA8, Sigma-Aldrich, Louis, USA), anti-CD68 (Maixin, Fuzhou, China), anti-MPO (anti-myeloperoxidase, Bio-techne, Minnesota, USA), anti-CD3 (Maixin, Fuzhou, China), and anti-CD20 (Maixin, Fuzhou, China) antibodies, respectively or successively. The TSA-RM 20U kit (Panovue, Beijing, China), a multiplex immunofluorescence tyramide signal amplification detection system, was used. Sections were treated with secondary antibodies (goat anti-mouse/rabbit-HRP) for 30 min at room temperature and incubated with fluorophore solution at dilution of 1:100. The cell nucleus was stained with 4′,6-diamidino-2-phenylindole (DAPI). Confocal Laser Scanning Fluorescence Microscope (Olympus Fv-3000, Japan) was utilized to collect fluorescence images.

### GSEA and GSVA analyses for IFI16

LN samples were divided into high and low expression groups based on the median values of IFI16 expression and assessed with GSEA and GSVA. The gene sets of “c5.go.bp.v7.4.symbols” from the MSigDB database were utilized to enrich the biological process pathways [36]. The GSVA algorithm was used to identify significantly enriched KEGG pathways between groups [37].

### Immune cell infiltration

The immunological characteristics of LN and control were examined by the CIBERSORT deconvolution algorithm. To exhibit the infiltration of 22 types of immune cells, differences in glomerular tissues between LN and control groups were visualized using a violin diagram created with R ggplot2 package [38]. Associations between IFI16 and immune infiltrating cells were further analyzed by Spearman’s rank correlation analysis and visualized using the ggstatsplot and “ggplot2 R packages.

### Statistical analysis

Statistical analyses were performed with SPSS (version 20.0, Chicago, IL, USA) and Prism 8 software (GraphPad, San Diego, CA, USA). Student’s t-test or one-way ANOVA for two or more independent samples were used to analyze differences in quantitative parameters among groups. Pearson’s test was used to evaluate correlations between parametric variables. Spearman’s test was used for nonparametric variables. To analyze prognostic risk factors, Kaplan–Meier curves were generated and compared by the log- rank test. *P* < 0.05 was considered significant.

## Results

### IFI16 is a potential biomarker for LN diagnosis by bioinformatics

**Fig. 1:**
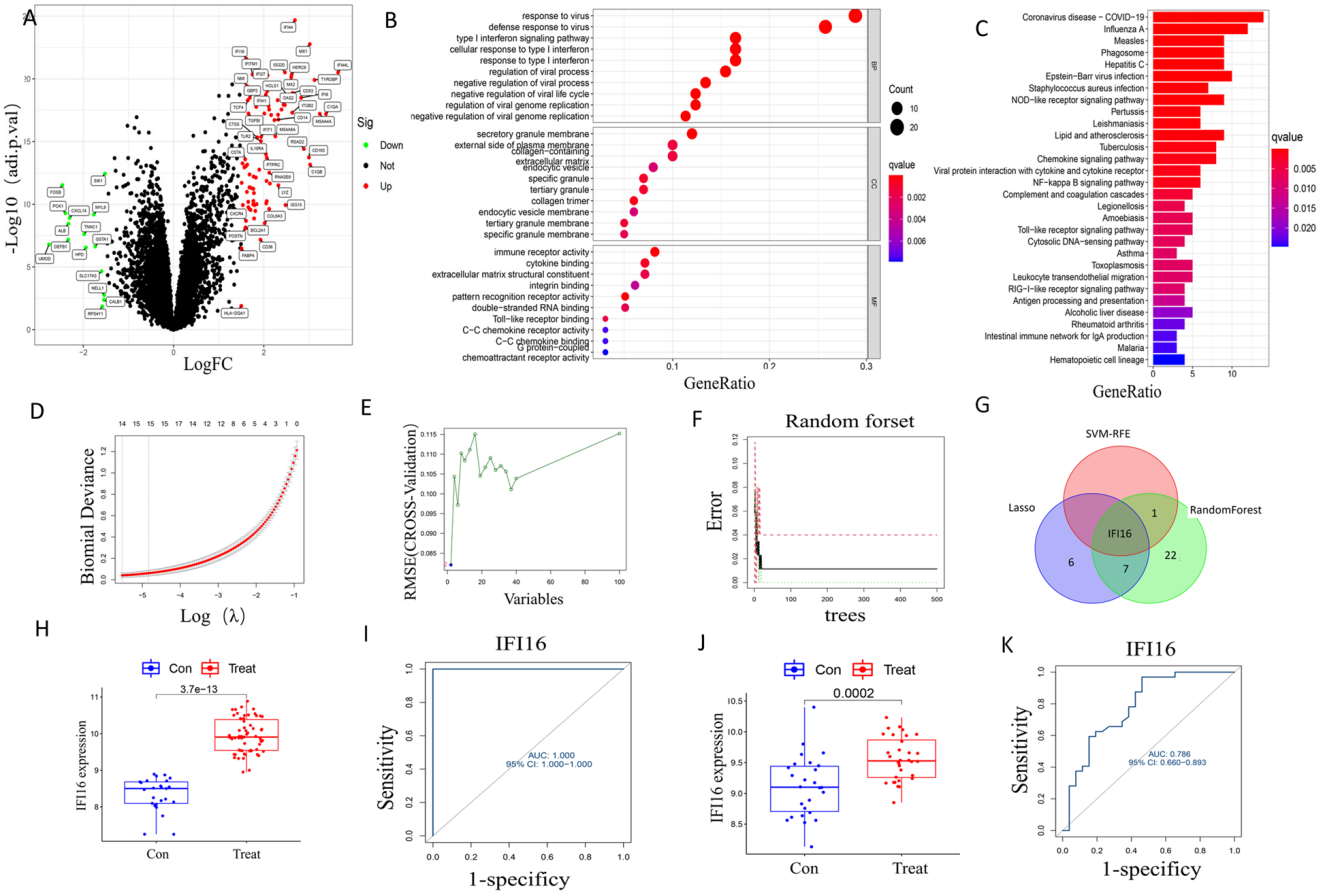
Bioinformatics analysis to identify key biomarkers in LN. **A** Volcano plots of the differentially expressed genes (DEGs). Red: genes upregulated in LN; green: genes downregulated in LN. **B** GO functional enrichment for the DEGs. **C** KEGG pathway enrichment for the DEGs. **D** Candidate diagnostic marker genes identified using LASSO logistic regression. **E** Candidate diagnostic marker genes identified using SVM-RFE. **F** Candidate diagnostic marker genes identified using RF algorithm. **G** Venn diagram of candidate diagnostic genes identified using three algorithms. **H** The difference in expression of IFI16 in training set. **I**  ROC curve for IFI16 using the training set. **J** The difference in expression of IFI16 in validation set. **K** ROC curve for IFI16 using validation set

LN patient samples (*n* = 62) and controls (*n* = 25) from 2 GEO data sets (GSE32591 and GSE99339) were re-analyzed. In total, 100 DEGs were identified: 85 were significantly upregulated, and 15 were downregulated (Fig. 1A). GO analysis of the DEGs indicated that immune-related processes were enriched (Fig. 1B). DEGs were mostly engaged in signaling pathways such as the NOD-like receptor, NF-kappa B, and Toll-like receptor signaling according to KEGG pathway enrichment (Fig. 1C).

LASSO logistic regression, SVM-RFE, and RF algorithms identified 14, 2 and 30 DEGs, respectively, as potential biomarkers (Fig. 1D-F). IFI16 was considered a good candidate for LN prediction as it was identified using all 3 algorithms (Fig. 1G). With respect to diagnostic efficiency, we mapped the expression of IFI16 in each sample and produced ROC curves. IFI16 expression was significantly higher in the LN group (*p* = 3.7 × 10^−13^) and successfully distinguished LN from NC, MCD, and DKD samples with AUCs of 1.000 (95% CI 1.000–1.000; Fig. 1H&I). IFI16 was also strongly expressed in the LN group (*p* = 0.0002 in the GSE104948 dataset which consisted of 32 LN patients and 26 controls (AUC of 0.786 (95% CI 0.660–0.893)), further validating its diagnostic efficiency (Fig. 1J&K). These results suggest that IFI16 expression has high potential for LN diagnosis.

### IFI16 is highly expressed in the kidney of LN patients

To further validate the expression of IFI16 in the kidney, we performed IHC staining on biopsy samples from 104 LN patients and 50 controls (including 12 MCD, 12 DKD, 12 IgAN and 14 NC samples). The general clinical information and pathologic data of recruited LN patients from the Frist Hospital of Jilin University were listed in Supplementary Table 1.

**Fig. 2:**
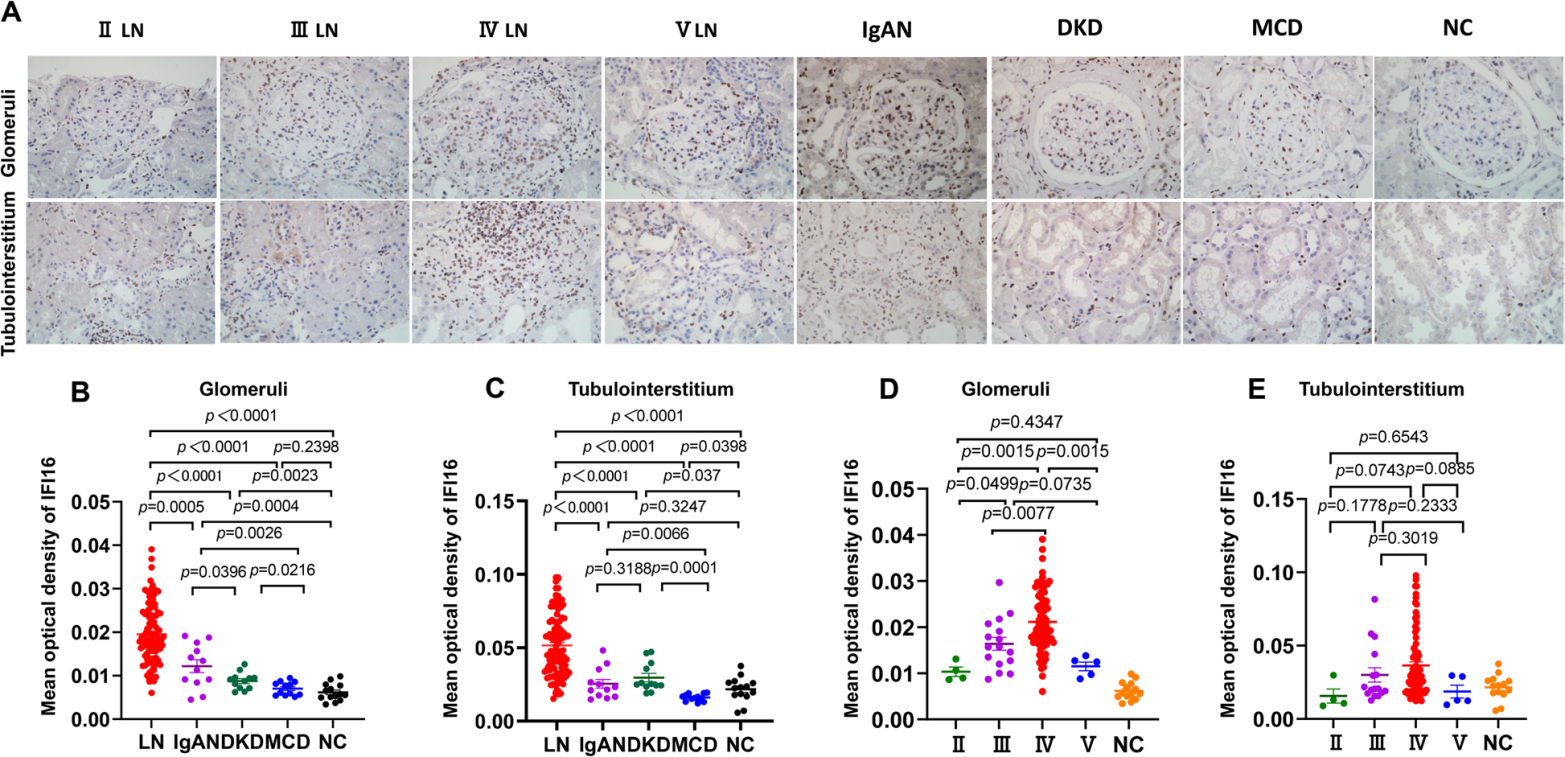
Immunohistochemistry staining of IFI16 in renal specimens. **A** Immunohistochemical staining of IFI16 in the glomeruli (above) and tubulointerstitium (below) of LN patients, DKD patients, MCD patients and NC (400x). **B** The mean optical density of IFI16 in the glomeruli and (**C**) tubulointerstitium. **D** The mean optical density of IFI16 in the glomeruli and (**E**) tubulointerstitium in different LN subclasses (class II, III, IV, V)

IHC showed that IFI16 was mainly expressed in the nuclei of cells in the glomerular and tubulointerstitial regions of the kidney in LN patients (Fig. 2A). In comparison to MCD, DKD, IgAN and NC samples, IFI16 expression was considerably greater in the glomerular and tubulointerstitial regions of LN patients (Fig. 2B&C). Regarding different pathological classes of LN, glomerular IFI16 expression was significantly higher in class IV LN patients compared to class II, III and V (Fig. 2D). The mean optical densities of the glomerular IFI16 were: class II: 0.0103 ± 0.002; class III: 0.0159 ± 0.0059; class IV:0.0212 ± 0.0072; class V: 0.0114 ± 0.0021(class II vs IV, *P* = 0.0015; class III vs IV, *P* = 0.0077; class IV vs V, *P* = 0.0015). However, no significant difference of IFI16 expression was found in the tubulointerstitium between different LN pathological class (Fig. 2E). These data indicate that glomerular IFI16 may be involved in the progression of LN.

### Colocalization of IFI16 expression with renal cells in LN patients

**Fig. 3:**
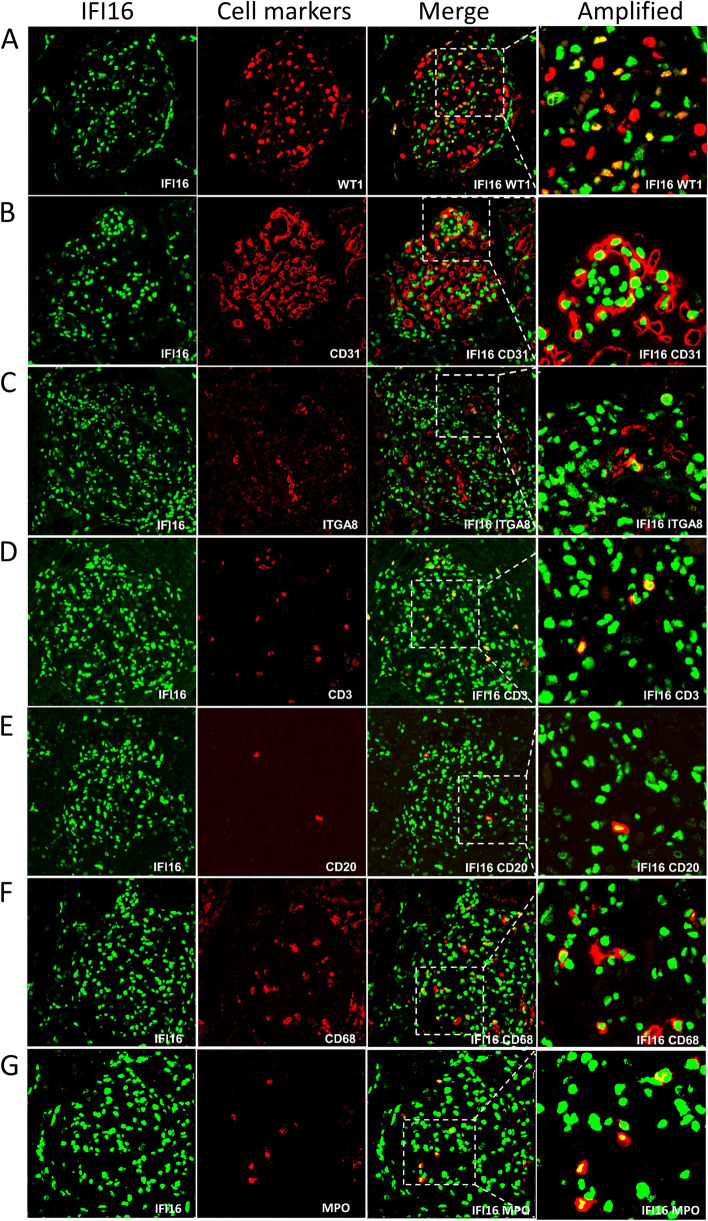
Multiplex immune fluorescence staining of glomerular IFI16 expression and renal cells in LN patients. Co-localization of IFI16 (green) with (**A**) WT1 (red) (marker of podocyte), **B** CD31 (red) (marker of endothelial cells), and **C** ITGA8 (red) (marker of mesangial cell). Co-localization of IFI16 (green) with **D** CD3 (red) (marker of T lymphocytes), **E** CD20 (red) (marker of B lymphocytes), **F** CD68 (red) (marker of monocyte cells), and **G** MPO (red) (marker of neutrophiles)

To examine the expression atlas of IFI16 and analyze its spatial relationships in various renal cell populations, multiplex immunofluorescence staining was carried out. IFI16 co-localized well with podocytes, mesangial cells, and glomerular endothelial cells in LN patients (Fig. 3). Additionally, IFI16 co-localized well with infiltrating inflammatory cells, including monocytes, T-lymphocytes, B-lymphocytes and neutrophils, in both glomerular and tubulointerstitial areas (Fig. 3, Supplementary Fig. 1). In normal renal tissues, IFI16 co-localized well with podocytes, mesangial cells and endothelial cells of the glomerulus, as well as co-localized well with the endothelial cells of tubulointerstitial area (Supplementary Fig. 2). These co-localization data indicate that both a variety of renal cells and inflammatory cells that infiltrate the kidney express IFI16.

### Clinicopathological significance of renal IFI16 expression in LN patients

**Fig. 4:**
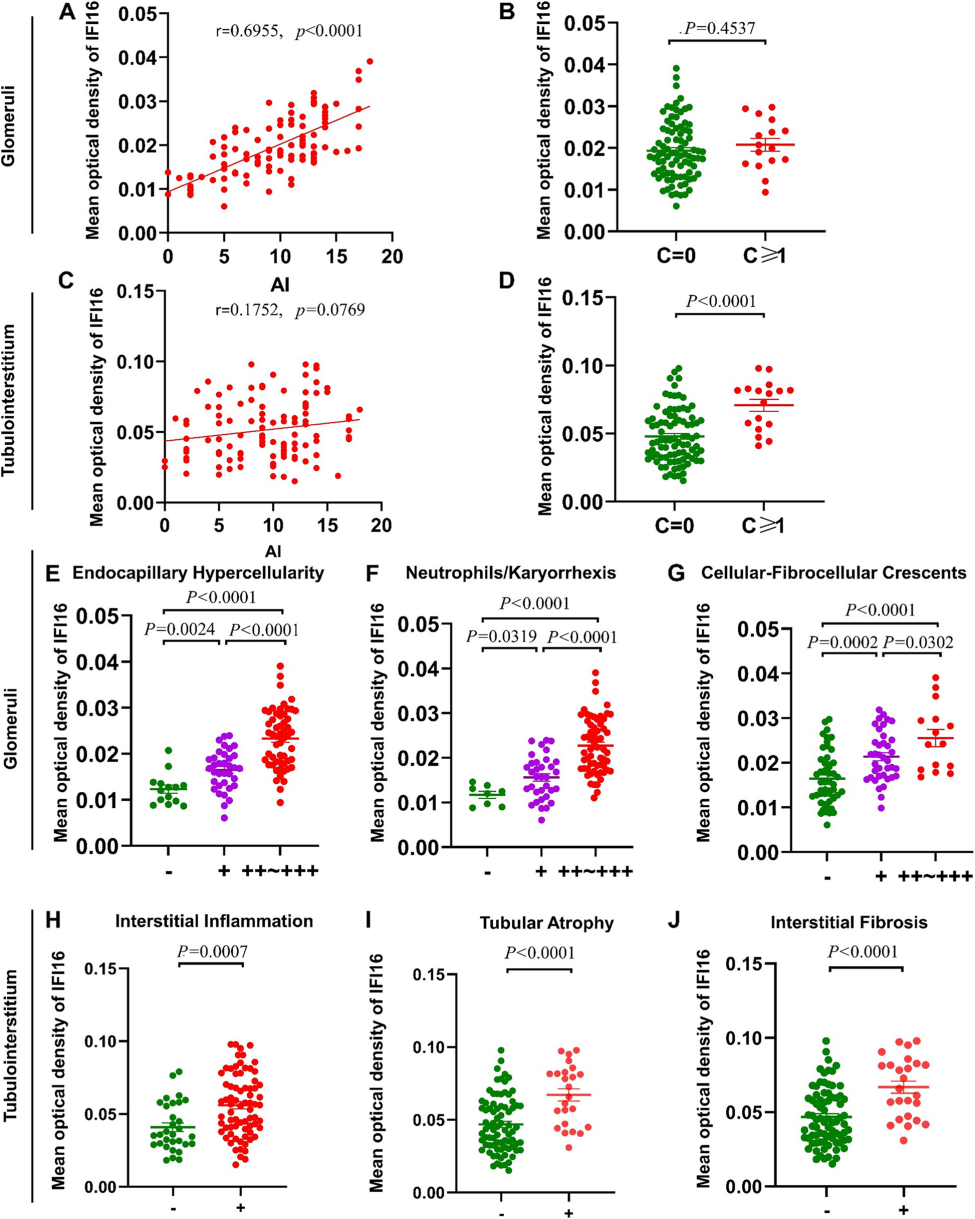
The association between IFI16 expression and renal pathologic indices in LN patients. The association of FI16 expression in glomerular area with (**A**) total AI and (**B**) total CI. Glomerular IFI16 expression with pathologic indices, including (**E**) endocapillary hypercellularity, (**F**) neutrophils/karyorrhexis, and (**G**) cellular-fibrocellular crescents. The association of FI16 expression in tubulointerstitial area with (**C**) total AI and (**D**) total CI. Tubulointerstitial IFI16 expression with pathological indices, including (**H**) interstitial inflammation, (**I**) tubular atrophy, and (**J**) interstitial fibrosis

To assess the clinicopathological significance of IFI16, we analyzed the relationship between renal IFI16 expression and severity of renal pathology. Glomerular IFI16 expression was positively correlated with the total pathological activity index (AI) (Fig. 4A; *r* = 0.6955, *P* < 0.0001) as well as several activity indices, including endocapillary hypercellularity, neutrophils/karyorrhexis, and cellular-fibrocellular crescents (Fig. 4E–G). However, no association between glomerular IFI16 expression and total chronicity index (CI) was found (Fig. 4B). Although there was no significant correlation between IFI16 expression in the tubulointerstitial region and total AI (Fig. 4C; r = 0.1752, *P* = 0.0769), there was a significant association between higher tubulointerstitial IFI16 expression and interstitial inflammation in LN patients (Fig. 4H; *P* = 0.0007). Additionally, IFI16 expression in the tubulointerstitium was substantially higher in the LN patients with renal chronicity (CI ≥ 1) than those without chronicity (CI = 0; Fig. 4D). Higher tubulointerstitial IFI16 expression was also seen in conjunction with interstitial fibrosis and tubular atrophy (Fig. 4I&J).

**Fig. 5:**
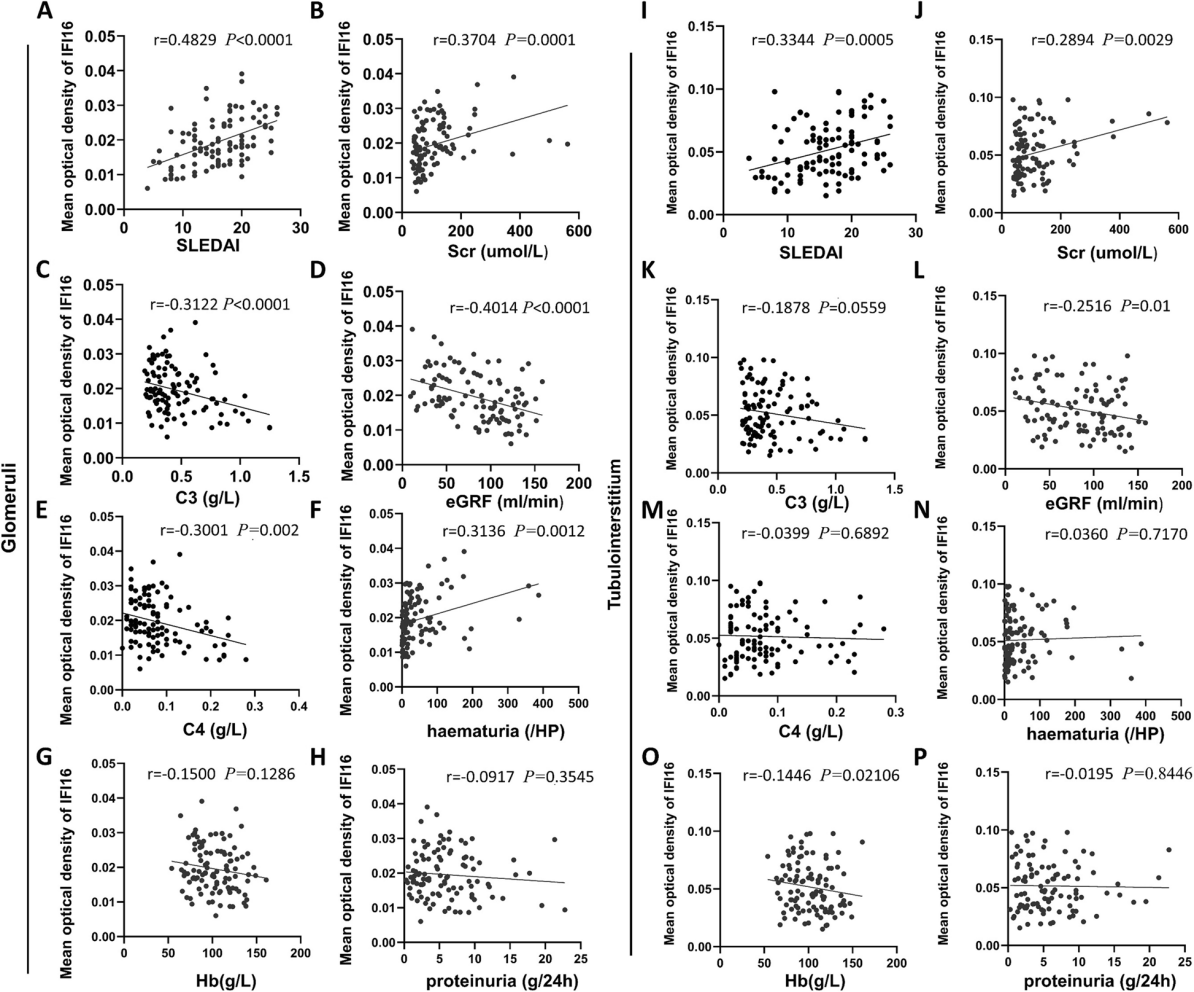
Correlations of IFI16 expression and clinical features in LN patients. Correlations of IFI16 expression in glomerular area with (**A**) SLEDAI, **B** serum creatinine, **C** serum C3, **D** eGFR, **E** serum C4, **F** hematuria, **G** hemoglobin, and **H** amount of 24-h urine protein in LN patients. Correlations of IFI16 expression in interstitial area with **I** SLEDAI, **J**serum creatinine, **L** eGFR, **K**, **M** serum levels of C3 and C4, **N** hematuria, **O** hemoglobin and **P** amount of 24-h urine protein

The associations between LN patient clinical parameters and expression of IFI16 in the kidney was also evaluated. Glomerular IFI16 expression was positively correlated with SLEDAI scores (r = 0.4829, *P* < 0.0001), serum creatinine (*r* = 0.3704, *P* = 0.0001), and the amount of hematuria (*r* = 0.3136, *P* = 0.0012), while negatively related to baseline complement C3 (*r* = -0.3122, *P* < 0.0001), C4 (r = -0.3001, *P* = 0.002), and eGFR (*r* = -0.4014, *P* < 0.0001) (Fig. 5A-K). For tubulointerstitial IFI16 expression, there was positive association with SLEDAI scores (*r* = 0.3344, *P* = 0.0005) and serum creatinine (*r* = 0.2894, *P* = 0.0029), negative correlation with eGFR (*r* = -0.2516, *P* = 0.01), and no obvious relationship with C3, C4, or the amount of hematuria (Fig. 5I-N). Additionally, LN patients were divided into two groups according to the mean optical density of IFI16 expression (The mean optical densities of IFI16 expression were: glomerular high group = 0.0252 ± 0.0048 and low group = 0.0141 ± 0.0048; tubulointerstitial high group = 0.0681 ± 0.0145 and low group = 0.0335 ± 0.0083). Obvious differences in the clinical characteristics of LN patients with high versus low IFI16 expression were observed (Supplementary Table 2). The results implied that higher renal IFI16 expression was associated with severe disease activity.

### Renal IFI16 expression is correlated with of LN patient prognosis.

**Fig. 6:**
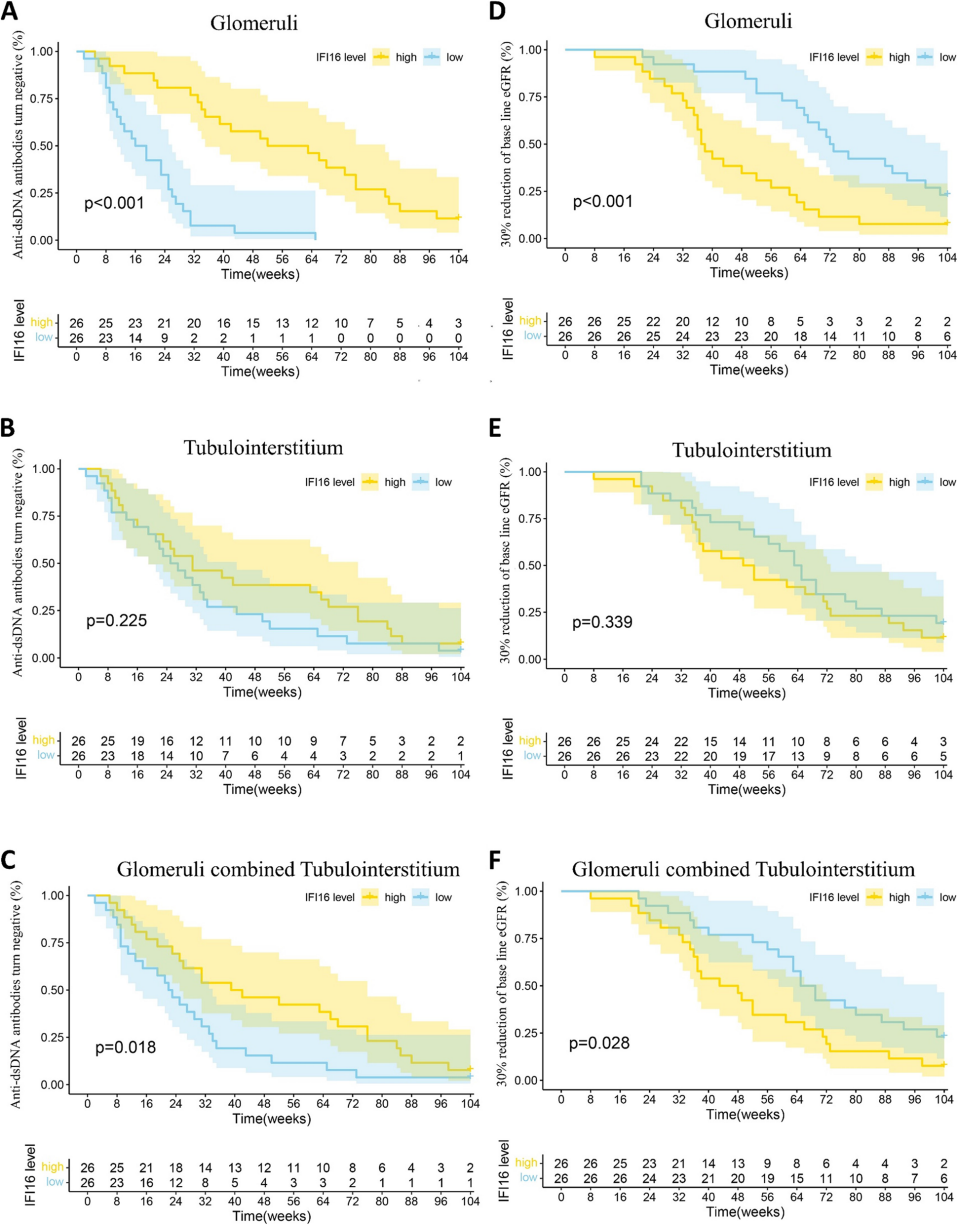
Association of IFI16 expression in kidney with prognosis of LN patients. Kaplan–Meier analysis of anti-dsDNA antibodies conversion from positive to negative between the high and low IFI16 expression groups in (**A**) glomeruli, **B** tubulointerstitium, and **C** glomeruli combined with tubulointerstitium. Kaplan–Meier analysis of 30% reduction of baseline eGFR between the high and low IFI16 expression groups in **D** glomeruli, **E** tubulointerstitium, **F** and glomeruli combined with tubulointerstitium

After excluding patients with incomplete follow-up data, a total of 52 patients were analyzed for prognosis. High IFI16 expression in the glomeruli or glomeruli combined with tubulointerstitium was a risk factor for prognosis and could predict a lower probability of anti-dsDNA antibodies conversion to negative as well as a higher probability of 30% reduction of baseline eGFR (Fig. 6A, C, D &F). Although there was no statistically significant IFI16 expression in the tubulointerstitium, there was a tendency for LN patients with higher IFI16 expression to have poorer prognosis (Fig. 6B&E). Based on multivariate Cox hazard analysis, IFI16 expression in the glomeruli as well as glomeruli combined with tubulointerstitium were both independent risk factors for the outcome after adjusting for age, sex, proteinuria, baseline eGFR and hypertension [anti-dsDNA antibodies conversion to negative: glomeruli (95% CI 1.84E-142, 5.63E-73), *P* < 0.001; glomeruli combined with tubulointerstitium (95% CI 1.01E-13, 0.5030), *P* = 0.040] [30% reduction of baseline eGFR: glomeruli (95% CI 2.85E + 13, 4.09E + 58), *P* = 0.0018; glomeruli combined with tubulointerstitium (95% CI 1.096, 9.59E + 10), *P* = 0.048]. These findings suggest that worsening prognosis in LN patients is related to increased IFI16 expression in the kidney.

### GSEA and GSVA: IFI16 is involved in the immunopathogenesis of LN

**Fig. 7:**
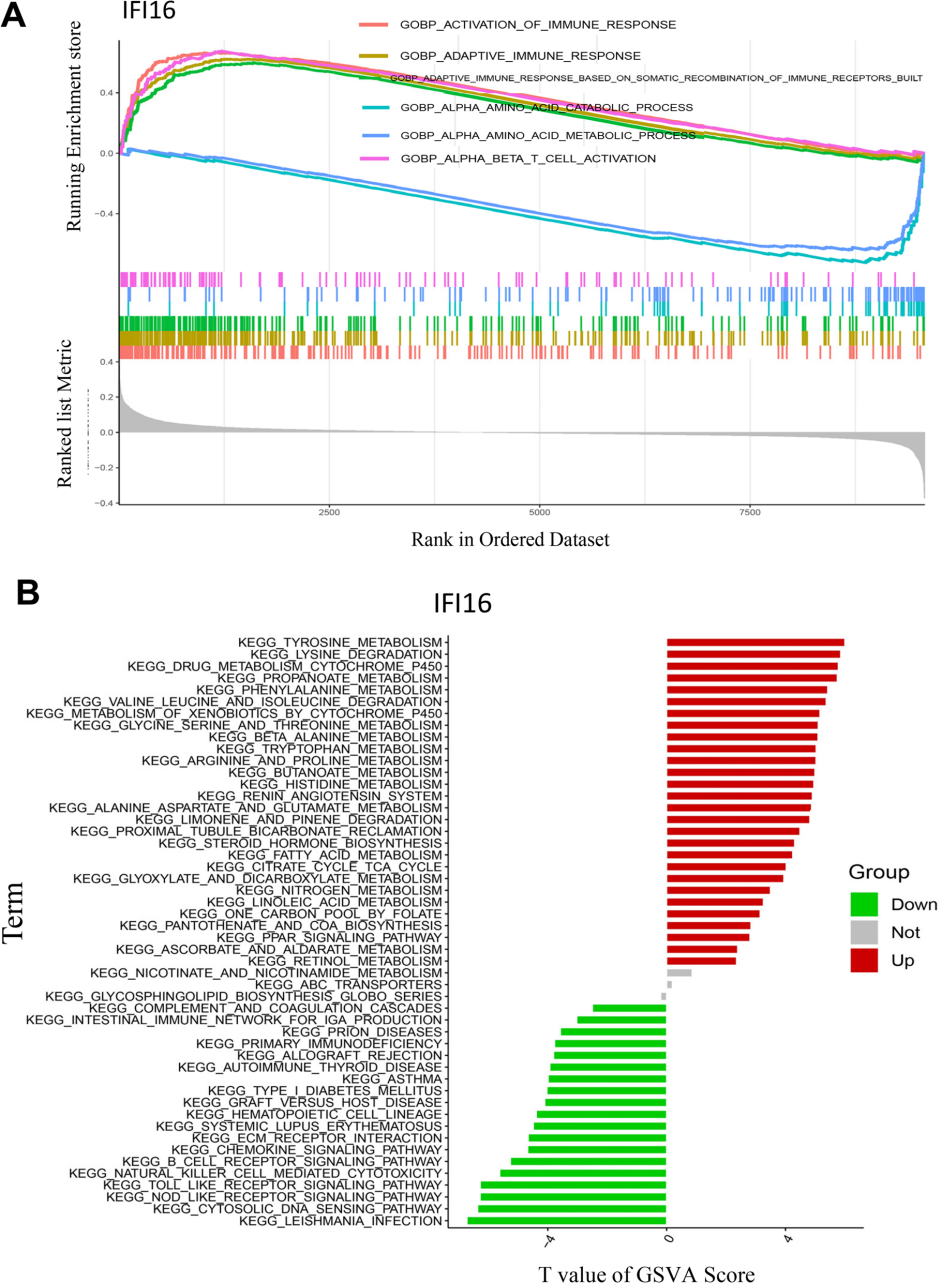
Biological processes and relevant mechanisms related to the IFI16 expression. **A** GSEA analysis for the biological processes related to IFI16. **B** GSVA analysis for the relevant mechanisms related to IFI16

To explore the potential mechanisms of IFI16 in the progression of LN, GSEA and GSVA analyses were carried out. GSEA results indicated obvious enrichment of activation immune response, adaptive immune response, and alpha/beta T-cell activation in patients with higher expression of IFI16 (Fig. 7A). In addition, GSVA analysis showed that higher IFI16 expression was closely associated with activation of several metabolic pathways (amino acids metabolism and fatty acid metabolism), and inhibition of certain signaling pathways, including Toll-like receptor, chemokine,and Nod-like receptor signaling (Fig. 7B). Both GSEA and GSVA results implied that IFI16 may contribute to the development of LN via immune-related processes.

### Immune-related factors are crucial for the involvement of IFI16 in LN

**Fig. 8:**
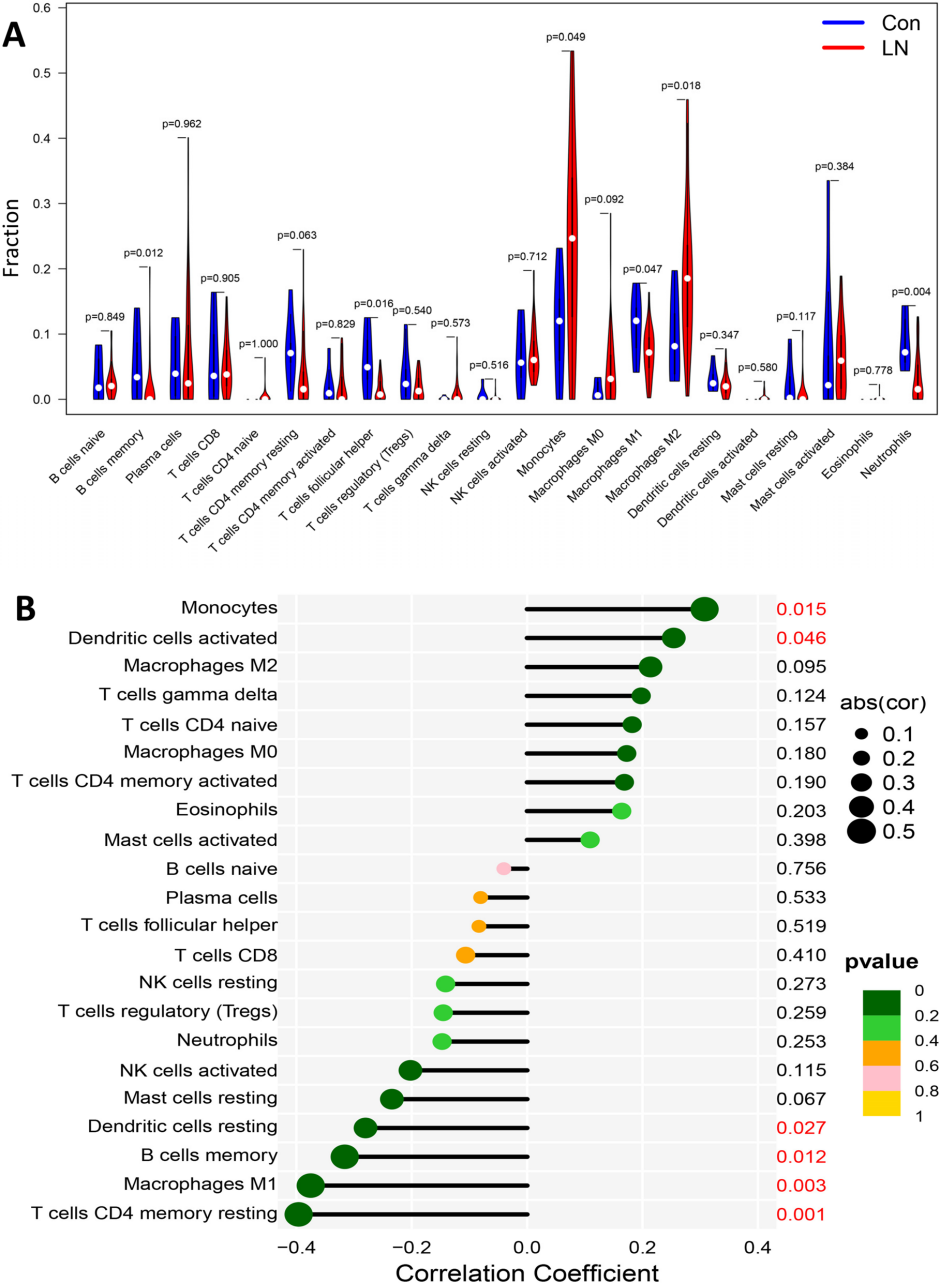
Analysis of immune cell infiltration. **A** Violin plot of immune cells with differential infiltration between LN patients and controls based on CIBERSORT. **B** Correlations between IFI16 expression and the extent of infiltration of immune cell subtypes

Considering that immune-related processes play crucial roles in the progression of LN, we investigated the immunological characteristics of LN through the CIBERSORT algorithm. Monocytes and M2 macrophages were more prevalent in the LN samples than in the control samples, while memory B cells, T follicular helper cells, M1 macrophages, and neutrophils were less prevalent (Fig. 8A). Notably, IFI16 expression had positive correlations with the infiltration of monocytes and activated dendritic cells, but negative correlations with resting dendritic cell infiltration, memory B cells, M1 macrophages, and resting T cell CD4 memory (Fig. 8B; r = 0.31, *P* = 0.015; r = 0.25, *P* = 0.046; r = -0.28, *P* = 0.027; r = -0.32, *P* = 0.012; r = -0.38, *P* = 0.003; r = -0.4, *P* = 0.001, respectively), implying the importance of immune-related factors for the engagement of IFI16 in LN.

## Discussion

Given the insidious symptoms at the early stage and lack of reliable predictors of disease progression, treatments for LN are still unsatisfactory nowadays [39]. Recently, bioinformatics combined with machine learning algorithms were utilized to discover potential diagnostic markers [10]. In this study, we first identified IFI16 as the candidate diagnostic marker of LN by conducting the abovementioned combined methods. Subsequently, expression of IFI16 was detected in the kidneys of LN patients through IHC technique. We found IFI16 expression in kidney biopsies from 104 LN patients. Renal IFI16, which was higher in LN patients, was significantly correlated with the severity of renal involvement. Specifically, glomerular IFI16 expression was mainly correlated with renal pathological activity indices, while tubulointerstitial IFI16 expression was mainly correlated with renal pathological chronicity indices. Meanwhile, higher levels of IFI16 were associated with higher SLEDAI scores, serum creatinine, as well as hematuria and lower baseline eGFR as wells as serum complements C3 and C4. Apart from that, higher IFI16 expression was closely related to worsening prognosis in LN patients. Our study provides novel insight into predicting LN clinical outcomes by renal biopsy.

IFI16 belongs to the interferon-inducible p200-protein family, which was initially detected in a wide variety of hematopoietic tissues [11, 40]. p200 proteins like IFI16 are constitutively detected in nonhematopoietic tissues, including the gastrointestinal tract, trachea, endothelial cells and stratified squamous epithelia of the skin and mucosae [41, 42]. In 2006, Mondini et al. pioneered the detection of IFI16 expression in skin tissues from individuals with SLE and systemic sclerosis [43]. They found that the expression of IFI16 was greatly increased and ubiquitous in all layers of the epidermis in lesioned skin from both patients with SLE and those with systemic sclerosis. In contrast, IFI16 was only found in the basal layer of the epidermis in healthy controls. In the same setting, dermal infiltrating inflammatory cells were positive for IFI16 staining. Vanhove et al. demonstrated that expression of IFI16 was increased in mucosa affected by active inflammatory bowel disease compared to normal mucosa [44]. Similarly, in the current study and for the first time to our knowledge, using IHC we detected IFI16 expression in renal biopsies from LN patients. Furthermore, multiplex IF staining showed that IFI16 co-localizes with certain renal cells and infiltrating inflammatory cells, indicating that IFI16 may contribute to the progression of LN via diversified mechanisms.

Biologically, IFI16 has been shown to down-regulate cell proliferation and increase apoptosis in several cell types. It was reported that IFI16 inhibits cell proliferation by impairing progression of the cell cycle at the G1-S phase transition [45]. Additionally, some studies suggest that IFI16 may act as an endogenous regulator of telomerase activity which contributes to inhibition of cell proliferation [45]. Furthermore, increasing studies have demonstrated that IFI16 collaborates with the p53 and p53-protein family members to stimulate the expression of the p21 gene and inhibit cell proliferation [46]. In our study, glomerular IFI16 in class IV, severe, diffuse proliferative LN was significantly higher than that in class III and II, focal, mild proliferative LN. In fact, in 2002, Gariglio et al. found high levels of IFI16 in stratified squamous epithelia with rapid proliferation. However, IFI16 is not uniformly expressed in stratified squamous epithelia; it is strongly detected in cells of the basal layer and gradually disappears as the cells migrate toward the superficial layer [41]. Thus, IFI16 expression in these rapidly proliferating epithelial cells as well as the higher IFI16 expression in severe proliferative LN in our study do not correlate with its capacity to inhibit cell proliferation, suggesting that IFI16 performs a function other than growth suppression.

GSEA results for our data indicated that high expression of IFI16 is enriched in both the active and adaptive immune responses as well as during alpha/beta T-cell activation. Meanwhile, the results of CIBERSORT algorithm showed that IFI16 expression positively correlated with monocyte infiltration and dendritic cell activated infiltration in the kidney. Considering the biological function of alpha/beta T-cells, monocytes, and dendritic cells, our GSEA and CIBERSORT results of high IFI16 expression enrichment during the adaptive immune response, alpha/beta T-cell activation, and monocyte and dendritic cell activated infiltration in the kidney suggest that renal IFI16 may be engaged in the local adaptive immune response in the patients with LN [47–49]. Additionally, comparative analysis of transcription profiles of activated T lymphocytes revealed significant upregulation of IFI16 expression in CD4 + and CD8 + T cells, suggesting a functional role of IFI16 in the activation of certain T cell subsets [50]. Similarly, we found that IFI16 co-localized with infiltrating inflammatory cells in LN renal tissues, including monocytes, T-lymphocytes and B-lymphocytes. This also supports the conclusion that IFI16 engages in the renal adaptive immune response in patients with LN. The mechanism of enhanced renal IFI16 expression and whether IFI16 could be a potential therapeutic target in LN patients need to be further studied.

Our present study has some limitations. First, it was only a single-center retrospective study. Second, the results of the prognostic analysis may be biased as a result of a relatively high percentage of patients being lost to follow-up. In light of this, we look forward to a further multi-center prospective study with more complete prognostic data to explore the role of IFI16 in LN.

## Conclusions

Renal IFI16 expression was associated with overall enhanced disease activity and worse prognosis in LN patients. Renal IFI16 could be a biomarker for disease activity and prognosis of LN patients, shedding light on predicting the renal response and the development of precise therapy for LN.

## Supplementary Information

**Additional file 1.** 

## Data Availability

The datasets presented in this study can be found in online repositories. The names of the repository/repositories and accession number(s) can be found in the article. Further inquiries can be directed to the corresponding authors.

## Abbreviations

LN: Lupus nephritis
SLE: Systemic lupus erythematosus
IHC: Immunohistochemistry
IF: Immunofluorescence
DKD: Diabetic kidney disease
MCD: Minimal change disease
NC: Normal controls
GSEA: Gene set enrichment analysis
GSVA: Gene set variation analysis
IFI16: Interferon-inducible protein 16
SLEDAI: Systemic lupus erythematosus disease activity index
anti-dsDNA: Anti-double-stranded DNA
AI: Activity index
CI: Chronicity index
NIH: National institutes of health
DEGs: Differentially expressed genes
GO: Gene ontology
KEGG: Kyoto encyclopedia of genes and genomes
RF: Random forest
SVM-RFE: Support vector machine-recursive feature elimination
LASSO: Least absolute shrinkage and selection operator
ROC: Receiver operating characteristic
AUC: Area under the ROC curve
ISN/RPS: International society of nephrology/renal pathology society
eGFR: Estimated glomerular filtration rate
anti-WT1: Anti-Wilms Tumor protein antibody
anti-ITGA8: Anti-integrin-ɑ8
anti-MPO: Anti-myeloperoxidase
DAPI: 4′,6-Diamidino-2-phenylindole
