# Supplementary material for: Renal interferon-inducible protein 16 expression is associated with disease activity and prognosis in lupus nephritis
Source: Arthritis Res Ther. 2023 Jul 1;25:112. doi: 10.1186/s13075-023-03094-8 (PMC10314472; doi:10.1186/s13075-023-03094-8)
Supplement: Supplementary file 1 — Additional file 1. [file 13075_2023_3094_MOESM1_ESM.docx]

**Supplemental Table 1: General clinical and pathological data of lupus nephritis patients**

| **Clinical information** | | **Laboratory Assessment** | | | **Renal Histopathology** | | |
| --- | --- | --- | --- | --- | --- | --- | --- |
| Sex (male/female), no. | 13/91 | | Leukocytopenia, no. (%) | 41 (39.42.) | | Classification |  |
| Age (years, mean+SD) | 35.38±11.64 | | Thrombocytopenia, no. (%) | 23 (17.30) | | II, no. (%) | 4(3.84) |
| Hypertension, no. (%) ^a^ | 38 (36.53) | | Low hemoglobin, no. (%) | 64 (61.53) | | III, no. (%) | 16 (15.38) |
| Renal function impaired, no. (%); | 34 (32.67) | | Hematuria, no. (%) | 87 (83.67) | | IV, no. (%) | 79 (75.96) |
| Nephrotic syndrome, no. (%) | 54 (51.92) | | Leukocyturia(noninfectious), no. (%) | 80 (76.92) | | V, no. (%) | 5 (4.81) |
| Fever (noninfectious), no. (%) | 28 (26.93) | | Proteinuria(g/24h), mean±SD | 6.03± 4.50 | | Activity indices score, median (range) | 9.40 (0-18) |
| Malar rash, no. (%) | 28 (26.93) | | Serum creatinine (µmol/L), median (range) | 109.28 (34.1~562.0) | | Endocapillary hypercellularity (+), no. (%) | 90 (86.53) |
| Oral ulcer, no (%) | 6 (5.76) | | Low serum C3, no. (%) | 97 (93.26) | | Cellular-Fibrocellular crescents, (+), no. (%) | 53 (50.96) |
| Alopecia, no. (%) | 14 (15.38) | | Low serum C4, no. (%) | 75 (72.11) | | Neutrophils/Karyorrhexis (+), no. (%) | 96 (92.30) |
| Arthralgia, no. (%) | 34 (32.69) | | ANA (+), no. (%) | 104 (100) | | Interstitial inflammatory cell infiltration, no. (%) | 73 (70.19) |
| Serositis, no. (%) | 16 (15.39) | | Anti-dsDNA (+), no. (%) | 87 (83.65) | | Chronicity indices score, median (range) | 0.48 (0-6) |
| Neurologic disorder, no. (%) | 4 (3.84) | | Anti- dsDNA (titer), median (range) | 1:32 (0 - 1:320) | | Glomerular sclerosis, (+), no. (%) | 19 (18.26) |
| SLEDAI (mean+SD) | 16.16±5.36 | |  |  | |  |  |

SLEDAI: systemic lupus erythematosus disease activity index. ANA: antinuclear antibodies. Anti-dsDNA: anti-double-stranded DNA antibody. mean+SD: mean+ standard deviation. a: Hypertension: blood pressure > 140/90 mmHg or the use of anti-hypertensive agents.

**Supplemental Table 2: Comparison of clinical characteristics of LN patients with high and low IFI16 expression**

| Clinical items Overall | | **Glomeruli** | | | | | **Tubulointerstitium** | | |
| --- | --- | --- | --- | --- | --- | --- | --- | --- | --- |
|  |  | High expression | | Low expression | | *P* | High expression | Low expression | *P* |
| Number | 104 | | 52 | | 52 | - | 52 | 52 | - |
| Male.no. (%) | 13 (15.39) | | 9 (17.31) | | 4 (7.69) | 0.236 | 7 (13.41) | 6 (11.53) | 1.000 |
| Age (yrs), mean ± SD | 35.38±11.64 | | 34.80±11.61 | | 35.96±11.75 | 0.799 | 37.21±11.81 | 33.56±11.28 | 0.110 |
| Serum creatinine (µmol/L), median (range) | 109.28 (34.1~562.0) | | 132.43 (41.0~562.0) | | 86.13 (34.1~375.0) | 0.008 | 127.87 (34.1~562.0) | 90.69 (38.7~275.9) | 0.034 |
| C3 (g/L), mean ± SD | 0.46±0.24 | | 0.39±0.15 | | 0.53±0.29 | 0.003 | 0.42±0.18 | 0.49±0.28 | 0.132 |
| Albumin (g/L), mean ± SD | 22.57± 6.52 | | 22.60± 6.04 | | 22.52± 7.03 | 0.950 | 22.47± 6.71 | 22.66± 6.39 | 0.882 |
| Proteinuria (g/24 h), mean ± SD | 6.03± 4.50 | | 5.92± 4.36 | | 6.14± 4.69 | 0.810 | 6.03± 4.69 | 6.02± 4.36 | 0.991 |
| SLEDAI, mean ± SD | 16.16±5.36 | | 18.05±4.97 | | 14.25±5.04 | 0.002 | 17.44±3.56 | 13.73±3.84 | ＜0.001 |
| Anti–double-stranded DNA antibody.no. (%) | 87 (83.65) | | 49 (94.25) | | 38 (73.07) | 0.006 | 42 (80.77) | 45 (86.53) | 0.596 |
| Hypertension, no. (%) | 38 (36.53) | | 18 (34.61) | | 20 (38.46) | 0.836 | 24 (46.15) | 14 (26.92) | 0.066 |
| Renal function impaired, no. (%) | 34 (32.67) | | 25 (48.07) | | 9 (17.30) | 0.001 | 19 (36.53) | 14 (26.92) | 0.399 |
| Hematuria, no (%) | 87 (83.67) | | 49 (94.23) | | 38 (73.07) | 0.006 | 47 (90.36) | 40 (76.92) | 0.109 |
| Leukocyturia (noninfectious), no. (%) | 80 (76.92) | | 45 (86.53) | | 35 (67.30) | 0.034 | 39 (75.00) | 41 (78.86) | 0.816 |

**Supplementary Figure 1**

**
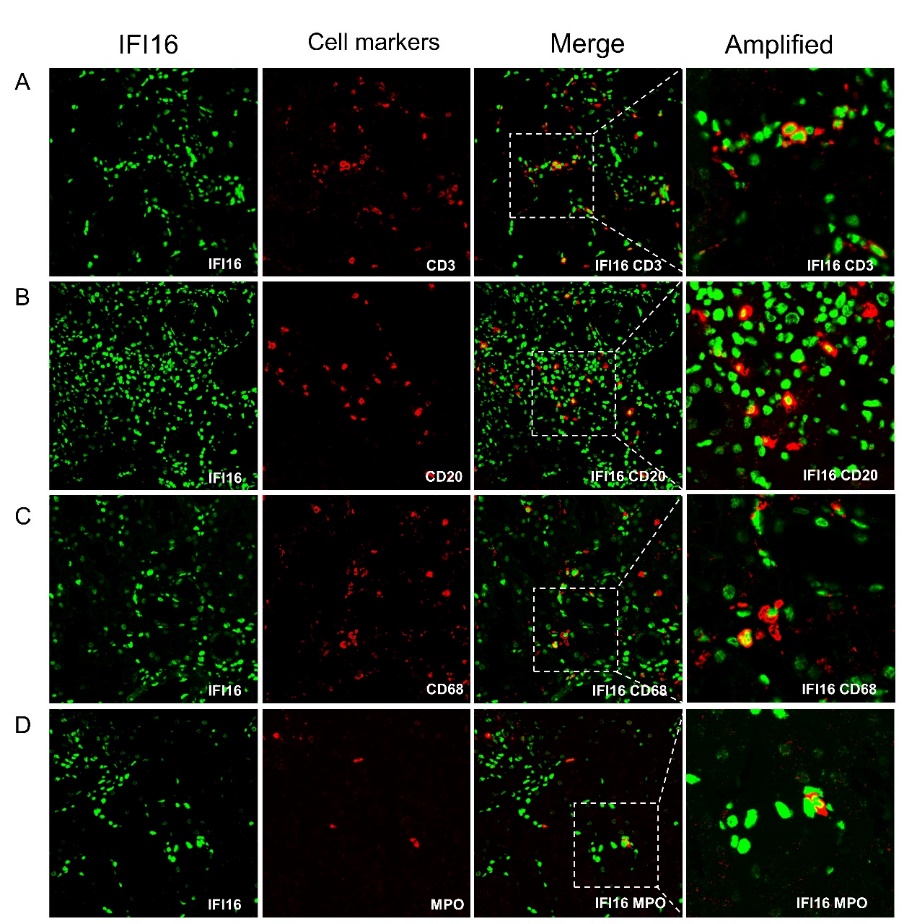
**

**Supplementary Figure 1. Multiplex immune fluorescence staining of tubulointerstitial IFI16 expression and cells in LN patients.**  Co-localization of IFI16 (green) with (A) CD3 (red) (marker of T lymphocytes), (B) CD20 (red) (marker of B lymphocytes), (C) CD68 (red) (marker of monocyte cells), and (D) MPO (red) (marker of neutrophiles).

**Supplementary Figure 2**


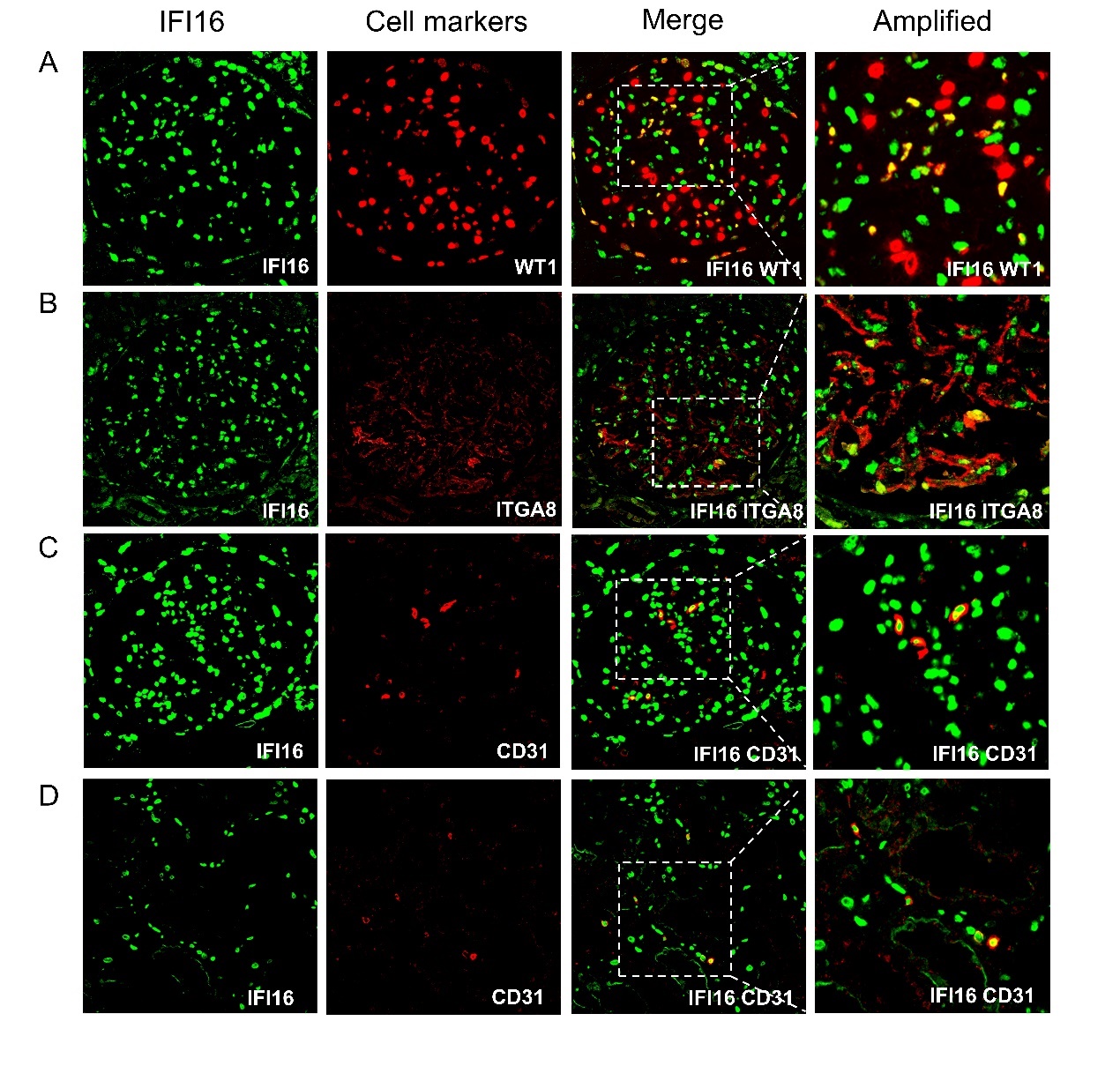


**Supplementary Figure 2. Multiplex immune fluorescence staining of IFI16 expression and renal cells in normal control.** In glomerulus, co-localization of IFI16 (green) and (A) WT1 (red) (marker of podocyte), (B) ITGA8 (red) (marker of mesangial cell), (C) CD31 (red) (marker of endothelial cells). In tubulointerstitium, co-localization of IFI16 (green) and (D) CD31 (red) (marker of endothelial cells).
